# Novel and Stress Relevant EST Derived SSR Markers Developed and Validated in Peanut

**DOI:** 10.1371/journal.pone.0129127

**Published:** 2015-06-05

**Authors:** Tejas C. Bosamia, Gyan P. Mishra, Radhakrishnan Thankappan, Jentilal R. Dobaria

**Affiliations:** 1 Crop Improvement Division, ICAR- Directorate of Groundnut Research, Junagadh, Gujarat, 362001, India; 2 Department of Biotechnology, College of Agriculture, Junagadh Agricultural University, Junagadh, Gujarat, 362001,India; National Key Laboratory of Crop Genetic Improvement, CHINA

## Abstract

With the aim to increase the number of functional markers in resource poor crop like cultivated peanut (*Arachis hypogaea*), large numbers of available expressed sequence tags (ESTs) in the public databases, were employed for the development of novel EST derived simple sequence repeat (SSR) markers. From 16424 unigenes, 2784 (16.95%) SSRs containing unigenes having 3373 SSR motifs were identified. Of these, 2027 (72.81%) sequences were annotated and 4124 gene ontology terms were assigned. Among different SSR motif-classes, tri-nucleotide repeats (33.86%) were the most abundant followed by di-nucleotide repeats (27.51%) while AG/CT (20.7%) and AAG/CTT (13.25%) were the most abundant repeat-motifs. A total of 2456 EST-SSR novel primer pairs were designed, of which 366 unigenes having relevance to various stresses and other functions, were PCR validated using a set of 11 diverse peanut genotypes. Of these, 340 (92.62%) primer pairs yielded clear and scorable PCR products and 39 (10.66%) primer pairs exhibited polymorphisms. Overall, the number of alleles per marker ranged from 1-12 with an average of 3.77 and the PIC ranged from 0.028 to 0.375 with an average of 0.325. The identified EST-SSRs not only enriched the existing molecular markers kitty, but would also facilitate the targeted research in marker-trait association for various stresses, inter-specific studies and genetic diversity analysis in peanut.

## Introduction

Peanut or groundnut (*Arachis hypogaea* L.), is generally cultivated in low-input farming systems, between 40° N and 40° S in the semi-arid tropical and sub-tropical regions of the world [1]. It is the sixth major oil-yielding, leguminous cash crop, which is cultivated in India approximately 20–25 million ha area, with a production of 35–40 million tons of pods annually [2]. It is a self-pollinated allotetraploid (2n = 4x = 40, AABB) crop having ten basic chromosomes with DNA content of about 2813 Mbp per 1C [3] with approximately 50000–70000 genes [4]. It belongs to genus *Arachis*, which is grouped into nine sections and includes approximately 80 species [5]. It is believed to have originated from a few or even a single hybridization event between *Arachis duranensis* (AA) and *Arachis ipaensis* (BB) [6], followed by spontaneous chromosome duplication [7].

Despite the high nutritional and economic implications; production and productivity is constrained due to various types of stresses, in most peanut growing areas of the world [1,8–11]. This crop is mostly grown under rain-fed conditions, where drought is a major constraint, limiting its productivity [11]. Similarly, salinity-affected area is approximately 830 million ha globally [12], an issue that deserves major attention. Marker-assisted selection is an important tool to enhance tolerance/resistance to these stresses and has the potential to enable faster and larger gains through genetic improvement [13]. Therefore, there is a need for the development of markers having functional relevance to various stresses in peanut.

In peanut molecular breeding programme, PCR based markers, like simple sequence repeats (SSRs) are considered as the marker of choice, due to many desirable attributes like co-dominance, high-variability, reproducibility, wide genome coverage and ease in use [14]. Peanut has comparatively lower genomic resources (including transcriptome data) compared to other legumes like medicago [15], lotus [16], and chickpea [17] which adds to the necessity of developing more robust molecular markers, specifically the genic ones, as they provide the insight into the functional information. Although, quite recently in April 2014, the genome sequence of two probable progenitors of cultivated groundnut, *A*. *duranensis* and *A*. *ipeansis* was completed with information available in public domain (http://peanutbase.org/), but the genome sequence of cultivated peanut, *A*. *hypogaea* is yet to be sequenced.

Development of SSRs using conventional experimental methods like SSR-enriched or non-enriched genomic library construction is quite laborious, time consuming and expensive. However, *in silico* development of Expressed Sequence Tag (EST) derived SSRs through mining of publicly available database are relatively simpler and cost effective [18,19]. Such EST-SSRs finds potential use in genetic research for molecular breeding in peanut. Alternatively, with the development of peanut based EST projects, vast amount of EST data has been generated which is available in the NCBI databases and offers an opportunity to identify SSRs [20,21]. However, the available peanut high throughput transcriptome sequences are not complete; many have low N50 values, ranging from 500 to 750 bp [22–25]. Because peanut has such a large number of genes, it is important to have a good representation of the transcriptome. On other hand, ESTs data of cultivated peanut is still remain unexplored for the development of SSR markers.

In peanut, work has been documented by various research groups on the development and characterization of both genomic SSRs [26–32] as well as genic SSRs [20,21,24,33–38]. However, nearly 10000 SSRs were available in peanut in public domain, which are grossly inadequate for genetic studies. Moreover, several peanut genetic maps have been published, but marker density is not satisfactory especially in the context of large genome-size and 20 linkage groups. Besides, the bottleneck with reference to paucity of polymorphic markers in cultivated peanut compared to the wild genotypes, makes an exclusive demand for the development of large number of markers for the effective molecular breeding in peanut [39,40]. Very recently, different success stories have started coming up, where a few SSR markers were validated and successfully used for the improvement of biotic stress resistance in peanut [10,41,42].

In the present study, large number of novel EST derived SSR markers have been developed and markers with functional relevance to different stresses have been validated, which will provide valuable resource for linkage mapping, integration of QTLs, comparative genomics and marker-assisted selection for improving peanut genotypes for various traits.

## Materials and Methods

All the experiments were done in the labs and fields of the Directorate of Groundnut Research and no animals were used

### Plant materials and DNA extraction

Eleven peanut genotypes, differing in their ability to various biotic and abiotic stresses, and also used as parental lines, for the development of various mapping population by different research groups, were used to screen the developed EST-SSR markers [43–47] (Table 1). The seeds of these genotypes were obtained from the Genetic Resources Section of the Directorate of Groundnut Research, Gujarat (India).

**Table 1:** List of parental genotypes used in current study along with its pedigree.

S. No.	Genotypes	Pedigree	Botanical types	Market type	Used as genotype or as parent in the cross for resistance studies	Remarks	Reference
1	GPBD4	KRG1 × CS16	Vulgaris	Spanish bunch	As cross with TAG24, TG26, GPBD5, TG19, TG49 and SG99	Cultivar, resistant to rust and late leaf spots (LLS), *Aspergillus* crown rot	10,14,43–46
2	JSP39 (GG16)	JSP14 × JSSP4	Hypogaea	Virginia runner	As genotype	Cultivar, tolerant to Peanut bud necrosis disease (PBND), stem rot and root rot diseases, thrips, S*podoptera* and leaf miner	43,44,47
3	R2001-3	ICG311 × ICG4728	Vulgaris	Spanish bunch	As cross with TG37A	Cultivar, resistant to rust and PBND; and tolerant to drought	10,43,44
4	ALR2	Selection from ICGV86011	Vulgaris	Spanish bunch	As genotype	Cultivar, resistant to LLS and rust	10,43,44
5	VG09405	CO3 x *A*. *kempffmercadoii*	Vulgaris	Spanish bunch	As genotype	Cultivar, resistant to rust	10,43,44
6	ICGV86590	X 14-4-B-19-B × PI 259747	Vulgaris	Spanish bunch	As cross with DH86, TG37A and JL24	Cultivar, resistant to multiple diseases (rust, LLS, PBND, stem and pod rots) and *Spodoptera litura*	10,14,43,44
7	NRCGCS85	(CT 7–1 × SB11) × *A*. *kretschmeri*	Vulgaris	Spanish bunch	As genotype	Inter-specific derivative, resistant to multiple diseases (PBND, stem rot, LLS, rust and alternaria leaf blight)	43,44,47
8	NRCGCS319	J11 x *A*. *duranensis*	Hypogaea	Virginia bunch	As genotype	Inter-specific derivative, resistant to stem rot	43,44,47
9	JL24	Selection from EC 95953	Vulgaris	Spanish bunch	As cross with ICGVSM 94584, ICGVSM 90704, ICGV86590, ICG11337, ICG(FDRS) 10	Cultivar, susceptible to multiple diseases including groundnut rosette disease and LLS	13,14,43,44
10	GG20	GAUG 10 × Robut 33–1	Hypogaea	Virginia bunch	As genotype and As cross with GPBD4 and ICGV86590	Cultivar, susceptible to multiple diseases and low aflatoxin contamination	10,43,44,47
11	TG37A	TG25 × TG26	Vulgaris	Spanish bunch	As cross with R2001-3	Cultivar, moderately tolerant to collar rot, rust and LLS, including drought tolerant	10,43,44

Two seeds of each genotype were grown in plastic pots filled with sand, under controlled conditions. Genomic DNA was extracted from fresh leaf tissue of one week old plants by CTAB method [48]. The quality of DNA was checked on 0.8% (w/v) agarose gel with λ DNA as standard and DNA was quantified using NanoDrop ND-1000 (NanoDrop products, DE, USA). The working concentration of DNA was adjusted to 20 ng μL^-1^.

### Data mining and processing of *Arachis hypogaea* ESTs sequences

From the NCBI database, 178490 EST sequences of *A*. *hypogaea* were downloaded (till 20^th^ March, 2013). Similarity search was done using all the SSR primer pairs sequence information available in the public domain. The candidate EST sequences were removed, using in-house Perl script (S1 Script). Remaining EST sequences were then processed for the removal of low-complexity regions which included trimming of poly- A, poly- T tracts, sequence ends rich in undetermined bases and low quality sequences (<100 bp). After that, NCBI UniVec databases (http://www.ncbi.nlm.nih.gov/VecScreen/UniVec.html) were used to detect potential vector contaminations, and were removed by Cross_Match software [49]. Finally, the remaining ESTs were assembled using TGICL software [50] using the command, tgicl<fasta_file>-p 95-l 50-v 20-s 10 000-O ‘-p 95-y 20-o 50’ [51] to reduce the sequence redundancy.

### Identification of microsatellites and functional annotation

For identifying the SSRs, the processed EST sequences were screened using MIcroSAtellite identification tool (MISA) [52] based on unit size or minimum number of repeats (2–6, 3–5, 4–4, 5–3 and 6–3) and the maximum number of bases interrupting two SSRs in a compound microsatellite = 100. The SSR containing sequences were separated by in-house Perl script (S2 Script) and subjected to online functional annotation tool, Blast2Go wherein BlastX of EST sequences (with e-value cut-off 10^–6^ or better) was carried out against the NCBI non-redundant protein sequence database (nr). Gene ontology terms were assigned to SSR containing sequences and visualized by online software WEGO [53] to understand the distribution of the gene functions.

### Primer designing

SSR containing EST sequences were used to develop EST-SSR primer pairs using online software BatchPrimer3 v1.0 [54]. The criteria used for primer designing were as follows primer length 18–23 bp, with optimum value 20 bp; T_m_ 57–63°C, with optimum value 60°C; GC content 40–60%, with the optimum value 50%; maximum Tm difference between forward and reverse primer 1.5°C and product size range 100–300 bp optimum value 150 bp.

### Screening and assessment of polymorphic EST-SSRs

The newly designed primers were further selected based on its relevance to various stresses and other functional unigenes. The selected primers were synthesized from Xcleris, India and polymerase chain reaction (PCR) was performed. The PCR mixtures (10 μl) contained 1μL template DNA (20 ng), 1 μL 10x Taq buffer + MgCl_2_ (15 mM), 0.8 μL dNTP (2 mM), 1.0 μL primers (10 p moles each, Forward and Reverse), 0.1 μL Taq polymerase (Promega 5U μL^-1^) and 5.1 μL sterile double distilled water. Amplification was performed in 0.2 mL (each tube) thin walled PCR plates (96 wells plate^-1^) in a thermal cycler (Eppendorf, Germany).

A touch down PCR amplification profile was programmed for 94°C for 3 min of initial denaturation, followed by first 5 cycles of 94°C for 30 s, 65°C to 60°C for 30 s and 72°C for 1 min, with 1°C decrement in annealing temperature per cycle, then 30 cycles of 94°C for 30 s with constant annealing temperature of 60°C and 72°C for 1 min followed by a final extension for 7 min at 72°C. Amplified products were analyzed using 6% non-denaturing poly-acrylamide gel at constant power 225 volts for about 2.5–3.0 h and stained with Ethidium bromide [10,55]. The gels were documented in automated gel documentation system (Fuji FLA-5000 PhosphorImager, Japan) and scored for the marker amplification.

### Data analysis

The size range of the amplified fragments for each microsatellite was estimated by using 50 bp DNA ladder (Fermentas, USA). Number of alleles and polymorphism information content (PIC) value for each polymorphic EST-SSR markers were determined using PowerMarker version 3.25 [56] The PIC was calculated as formula $PIC\;=\;1-\sum {P_{i}}^{2}-\sum_{i}.\sum_{j>i}2{P_{i}}^{2}{P_{j}}^{2},$ where Pi and Pj are the frequencies of the i^th^ and j^th^ alleles, respectively [57].

## Results and Discussion

### Sequence data assembly by TGICL program

Redundancy of SSR markers developed from different research groups along with the use of non-uniform marker names have resulted in duplicate genotyping of peanut germplasm and inefficient use of resources for peanut genomics [58]. Thus, development of non-redundant novel set of markers which excludes publically available SSR markers could assist in enhancing such efforts towards generation of genomic resources. In this context, an attempt has been made to develop non-redundant markers from ESTs database available from the public databases. Analysis of all publicly available *Arachis hypogaea* ESTs (178490 numbers) indicated a huge variation in length of sequences which ranged from 37 to 2038 bp with an average of 571 bp.

#### Non-redundant sequence assembly

In order to find novel non-redundant EST-SSR markers, all the publically available SSR primer sequences [20,21,24,26,27,29,34,35,37,58–60] were subjected to sequence similarity search with available EST sequences and 23,696 (13.28%) very similar sequences were excluded from further analysis. This step contributed to the identification of novel SSR markers not yet reported in the peanut. Prior to EST assembling, remaining sequences were subjected to pre-processing *viz*., removal of vector contamination, low-complexity sequences of less than 100 bp and poly A/T sequences. Pre-processing reduced the overall noise in EST data and thus improved the efficacy of subsequent analysis [61].

The sequences downloaded from NCBI displayed huge variation in its length with an average length of 571 bp, which indicated considerable amount of long transcripts. Assembling the ESTs constitutes an important step to provide non-redundant and high-quality sequences for the development of SSRs [61]. The long as well as high quality transcripts, totaling 138628 were assembled using TGICL software (Table 2). The TGICL software is quite efficient in handling long reads or transcripts [50] and there have been several reports of using TGICL for clustering the EST transcripts [17,38]. Moreover, TGICL assembly was also used to reduce the redundancy present in the publically available ESTs [62]. In the present study, TGICL assembly reduced the redundancy by 82.21% and generated unigenes with an average length of 857 bp and contig N50 length of 942 bp.

**Table 2:** Summary and statistics of *Arachis hypogaea* ESTs assembled by TGICL program at stringency of 50 bp similarity and 95% identity.

Features	Values
EST sequences available at NCBI	178490
ESTs removed based of primer sequence similarity	23696 (13.28%)
High quality ESTs utilized for assembly	138628 (77.67%)
Total number of unigenes	16424
Average length of unigene sequences (bases)	857
Number of contigs	13429 (81.76%)
Average number of ESTs in contigs	10.3
N50 contigs length (bases)	942
Numbers of singletons	2995 (18.24%)
Redundancy removed after assembly	82.21%

The N50 value is a statistical measure of average length of a set of sequence, which is used especially in reference to contig length. Within an assembly, it was found considerably higher than earlier report of 823 bases [23] whereas, similar results were reported by Chen et al. [63] with an average read length of 589 bp and N50 length of 974 bp, while assembling the long transcripts. This means, pre-processed long EST transcripts were handled quite efficiently by the TGICL assembler. Higher values of N50 and average read length of clustered datasets, provides longer lengths of contigs for selection of SSR flanking sequence, thus assisting in efficient designing of SSR primers [64]. In terms of *de novo* transcriptome assembly, TGICL performs better than any other assembler as it was developed especially for assembling the long EST reads [50]. Corroborating with the same, Bräutigam et al. [65] also judged TGICL as one of the best assembler in terms of contig length, hybrid assemblies, redundancy reduction and error tolerance in the study of non-model C_3_ and C_4_ crops.

### Identification and characterization of EST- SSR motifs

Out of 16424 unigenes subjected for SSR screening, 2784 (16.95%) SSR containing unigenes were identified harboring 3373 SSR motifs. The sequences harboring more than one SSR and compound SSRs were 17.49% (487) and 10.38% (289) respectively with an average frequency of one SSR per 4.17 Kb (Table 3). The density of SSR containing sequences was higher in the present study than previous reports in peanut *viz*., 13.34% [24] and 12.41% [21]. The frequency of SSRs obtained was also consistent with the frequency range of 2.65 to 10.62%, which has been reported in 49 dicot species [66]. Moreover, the frequency of EST-SSRs is known to be significantly influenced by the factors like repeat length and the criteria used for SSRs mining [67].

**Table 3:** Feature of microsatellites identified by MISA in non redundant EST sequences of *Arachis hypogaea*.

Feature	Values
Total number of sequences examined	16424
Total size of examined sequences (Mb)	14.08
Total number of identified SSRs	3373
Number of SSR containing sequences	2784
Number of sequences containing more than one SSR	487 (17.49%)
Number of SSRs present in compound formation	289 (10.38%)
Average frequency of SSRs(Considering total bases of 14.08 Mb)	1/4.17 kb
Total number of sequences annotated	2027 (72.81%)
Without mapping results	243 (8.73%)
With Blast results	193 (6.93%)
Number of sequences without Blast hits	320 (11.49%)

Annotation of SSR containing sequences was done at e-value ≤ 10^–6^

In earlier reports, frequency of SSR motifs in *Arachis hypogaea* was observed in the range of one SSR per 4.52 to 7.3 kb [21,37,38]. However, in this study, it is slightly less (one SSR per 4.17 Kb) which reflected relatively high density of SSRs in the EST sequences. The frequency of SSRs in other legumes, like chickpea and Medicago is reported as one SSR per 8.54 kb [68] and 7.47 kb [69] respectively. However, any sort of direct comparison about abundance and frequency of SSRs with other reports is very difficult, since the estimates were dependent on various factors like SSR search criteria, size of the dataset, database mining tools and the EST sequence redundancy [67,69].

### Functional annotation of unigene containing SSRs

The SSR containing unigenes were subjected to functional annotation by Blast2GO software so as to find its putative function(s). A total of 2463 (88.51%) unigenes matched with BlastX search of nr proteins database with e-value cut-off 10^–6^ or better, of which 2027 (72.81%) SSR containing ESTs were fully annotated for functional protein-encoding sequences, whereas 756 (27.19%) were either putative or hypothetical or uncharacterized or unknown or with no considerable homology (Table 3). The uncharacterized transcripts can be attributed to the unavailability of fully annotated peanut genomic data, and also to the limited numbers of characterized transcripts in this crop. Partially, it can also be attributed to the lack of information with respect to number of protein-encoding genes and transcripts derived from alternative splicing in peanut [63]. However, the percentage of uncharacterized transcripts was very less, compared to 52.77% of sequences having no significant match in study of whole plant transcriptome of *A*. *hypogaea* Spanish botanical type [70]. Hence, it can be assured that most of the sequences harboring SSRs were annotated using Blast2GO functional annotation tool by and maximum data was utilized.

**Fig 1:**
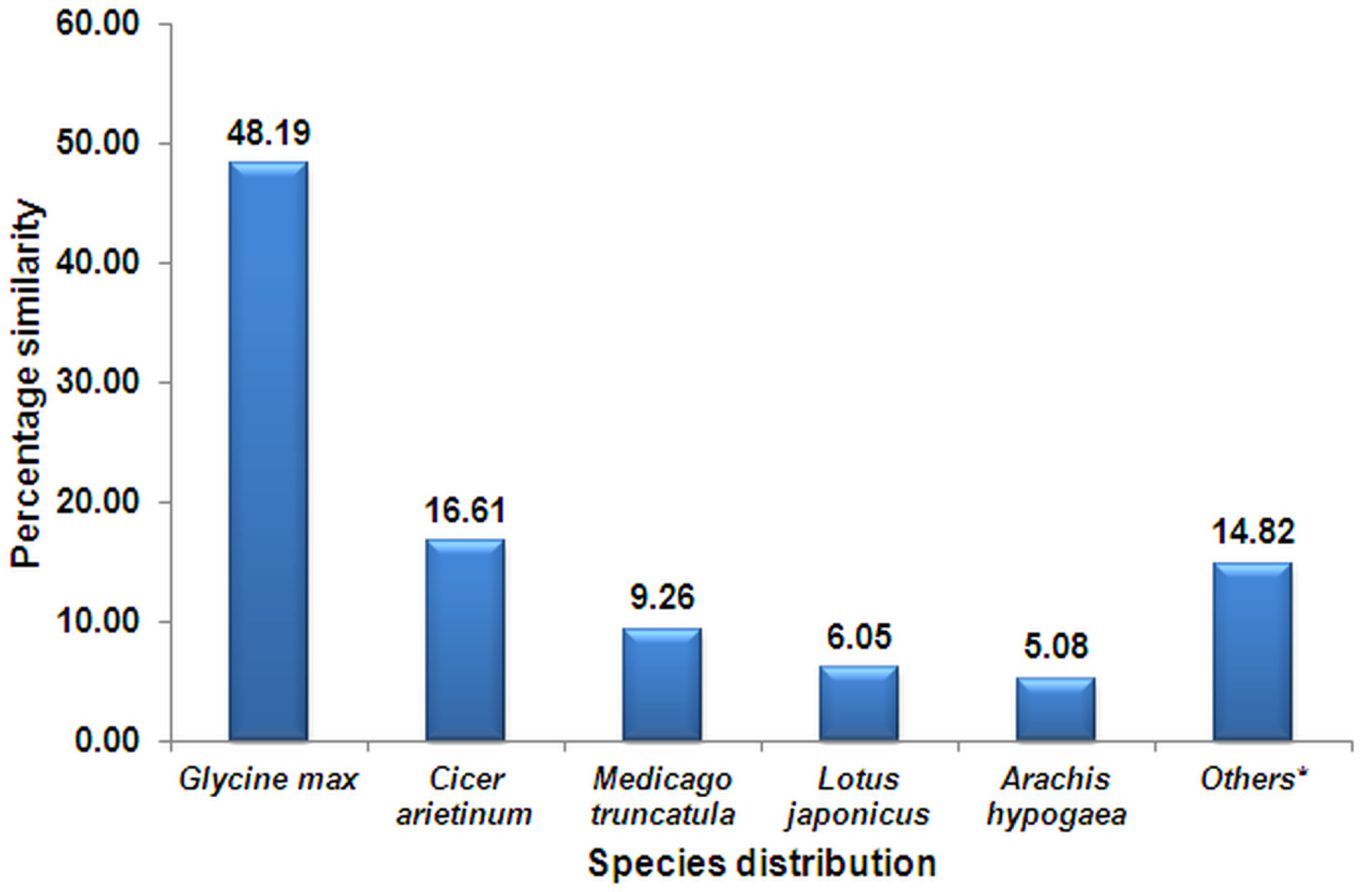
Distribution of best Blast hits for *Arachis hypogaea* ESTs-SSR containing sequences against other species. (*Others constitute the species having <2% similarity in Blast hit)

The best blast hit distribution revealed 48.2% sequences having similarity with *Glycine max* followed by *Cicer arietinum* (16.6%) and other legumes (Fig 1). On the other hand, the best blast-hits distribution with *Arachis hypogaea* contributed only 5.08% sequence similarity, which is mainly due to the less number of genes identified and characterized in peanut, compared to other legumes like *Glycine max* or *Cicer arietinum* [63]. The results were in agreement with the conservation of SSR loci and high level of synteny across the legumes [13,71].

#### Assignment of Gene Ontology (GO) terms

**Fig 2:**
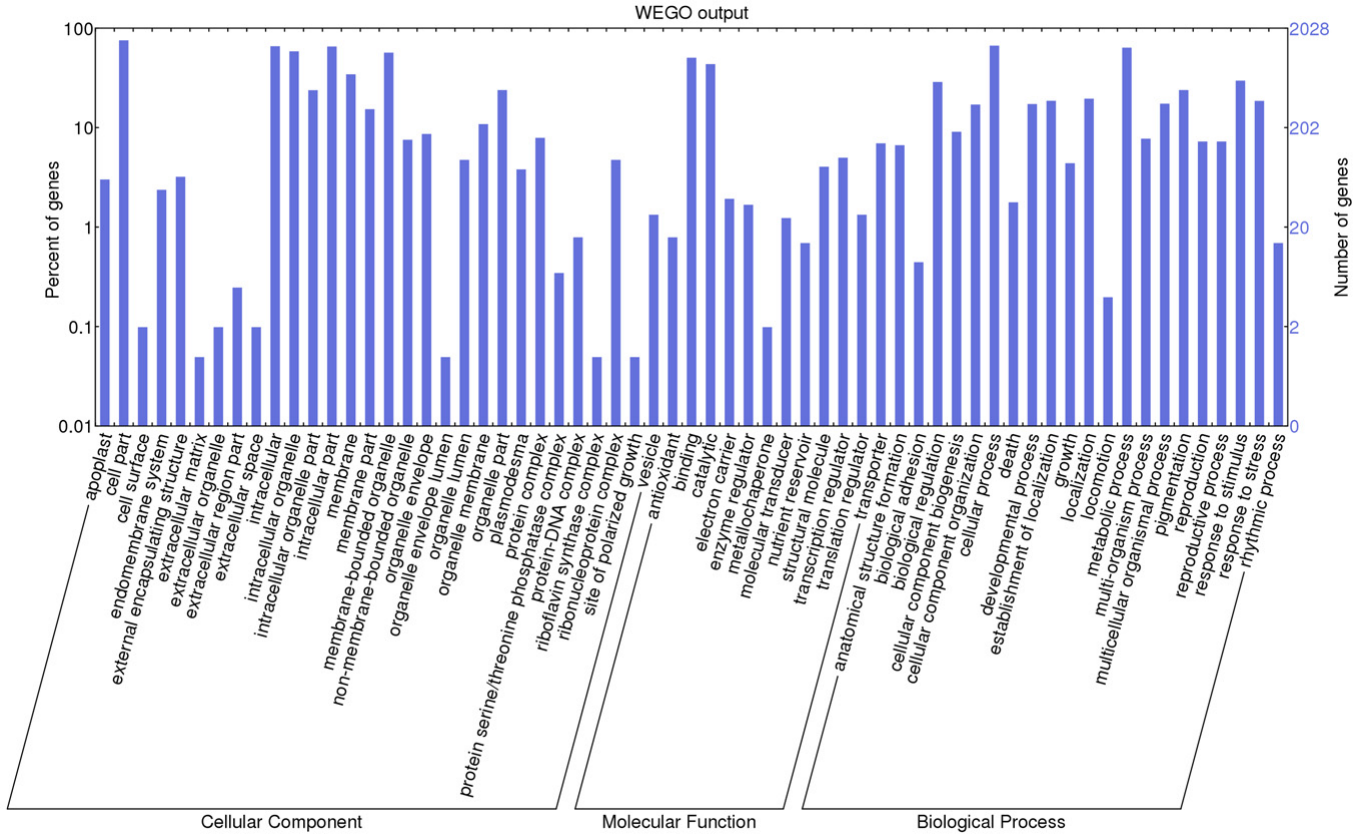
Distribution of most abundant Gene ontology (GO) terms assigned to 2027 annotated SSR containing sequences.

On the basis of gene annotations, of 2784 SSR containing sequences, 2027 (72.81%) were annotated and assigned in 4124 gene ontology (GO) terms, which are further categorized into biological ontology of cellular components (391), molecular functions (1120) and biological processes (2613). Distribution of GO term in cellular components, molecular functions, and biological processes revealed the maximum association with cell part (GO: 0044464), binding genes (GO: 0005488), and cellular process (GO: 0009987) respectively (Fig 2). Similar proportions of the biological ontology were also observed in the study of transcriptome analysis during seed development in peanut [23]. Additionally, 31.91% sequences were found to be associated with response to stimulus (GO: 0050896) encompassing biotic as well as abiotic stresses. The gene ontology categorization of EST sequences harboring SSR is represented in the Fig 2.

### Abundance and distribution of EST-SSRs motifs

**Fig 3:**
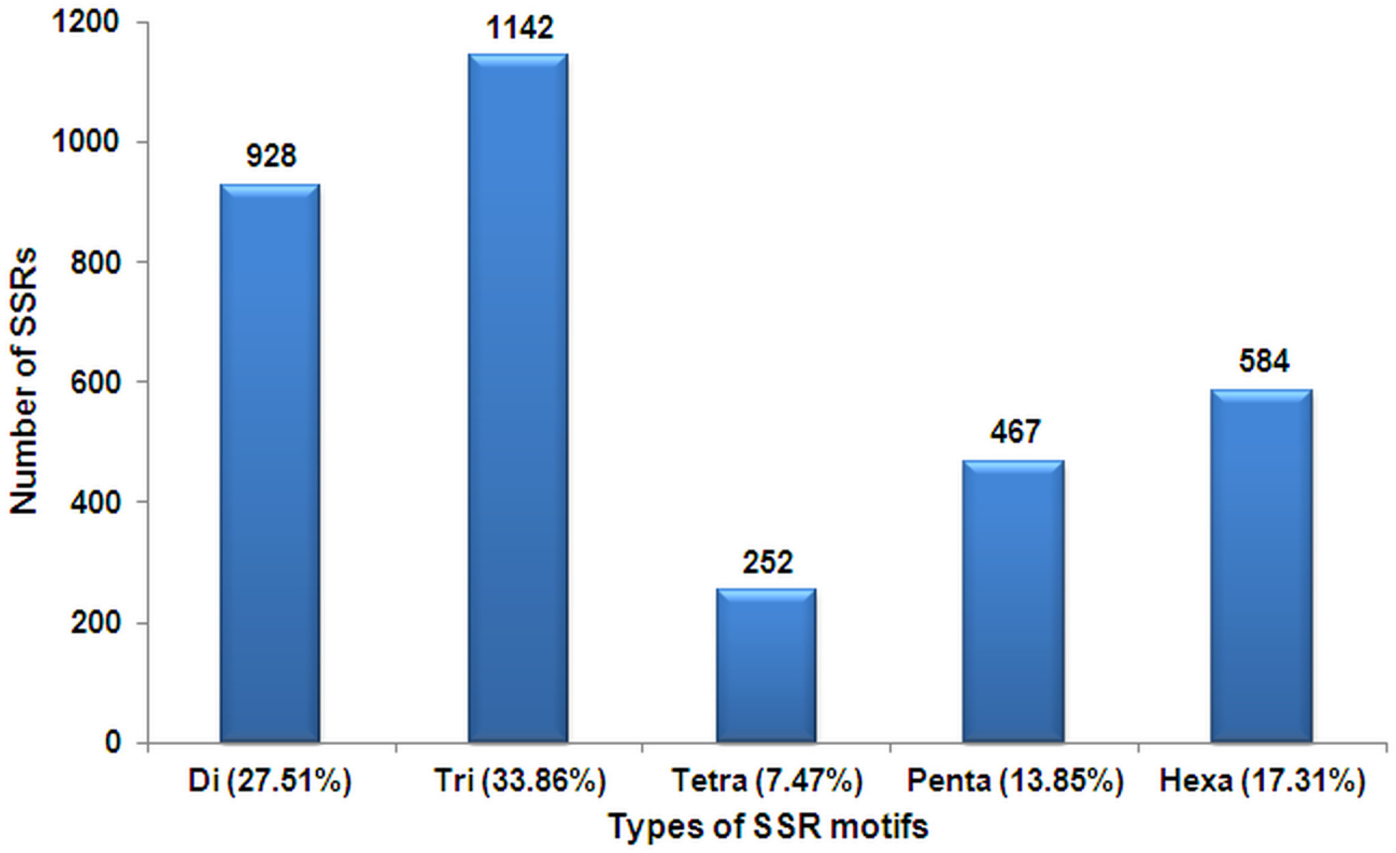
Distribution of 3373 EST-SSRs motifs based on MISA script.

Among 3373 SSR motifs, tri-nucleotide motifs were found most pre-dominant (33.86%) followed by di-nucleotide repeats (27.51%). Tetra-, penta- and hexa- nucleotides repeats recorded 7.47%, 13.85%, 17.31% frequencies respectively (Fig 3). As reported earlier, tri-nucleotide repeats were generally most abundant in SSR markers of both dicots and monocots [67,69]. The tri-nucleotide repeat abundance in the present study also corroborated with the earlier studies in peanut [20,21,24] and other legumes like medicago [69], chickpea [68] and field-pea [72]. The abundance of tri-nucleotide repeat motifs is quite common for EST-derived SSRs, as additions or deletions within translated regions, mostly do not disturb the open reading frames (ORFs), and thus can be tolerated more over other types of repeats [72,73]. It is also very well shown by Metzgar et al. [74] that in exons, trinucleotide repeats are invariably the most abundant in all taxa. Moreover, 6 bp di-nucleotide repeats comprised highest (30.6%), among different types of di-nucleotide repeats (Table 4). Like tri-nucleotide repeats, this combination also does not alter the ORF, largely at the functional level, thus favoured and retained by the system [73]. Among the di-nucleotide repeats, AG/TC (75.2%) was the most abundant while AT/AT and AC/GT motifs accounted for 15.4% and 9.4% respectively (Table 4).

**Table 4:** Frequency distribution of di—and tri- nucleotide motif repeats in peanut.

Di-nucleotide	Number of repeat motifs								
	5	6	7	8	9	10	>10	Total	Percentage
AC/GT	0	52	12	12	7	1	3	87	9.4
AG/CT	0	170	122	91	64	52	199	698	75.2
AT/AT	0	62	32	17	7	4	21	143	15.4
**Total**	**0**	**284 (30.6%)**	**166 (17.9%)**	**120 (12.9%)**	**78 (8.4%)**	**57 (6.1%)**	**223 (24.0%)**	**928**	**-**
**Tri-nucleotide**									
AAG/CTT	202	124	45	30	20	9	17	447	39.1
AAT/ATT	86	34	20	10	2	1	9	162	14.2
ACC/GGT	46	22	16	6	1	0	1	92	8.1
ACG/CGT	7	4	1	0	1	0	0	13	1.1
ACT/AGT	10	1	2	1	0	0	1	15	1.3
AGC/CTG	40	14	4	2	2	0	0	62	5.4
AGG/CCT	44	20	2	5	1	0	0	72	6.3
ATC/ATG	90	35	17	8	5	5	3	163	14.3
CCG/CGG	20	8	1	0	0	0	0	29	2.5
**Total**	**590 (51.6%)**	**286 (25.04%)**	**117 (10.2%)**	**65 (5.7%)**	**36 (3.2%)**	**15 (1.3%)**	**33 (2.9%)**	**1142**	**-**

Of the tri-nucleotide repeat motifs, AAG/CTT (39.1%) was the most abundant followed by ATC/ATG (14.3%) and AAT/ATT (14.2%) (Table 4). The abundance of AAG repeat affirms the earlier report of Zhao et al. [58]. The AT-rich tri-nucleotide motif (AAG/CTT, AAT/ATT, ACT/AGT, and ATC/ATG comprised ~70%) were more abundant than GC rich tri-nucleotide motif (ACC/GGT, ACG/CGT, AGC/CTG, AGG/CCT and CCG/CGG comprised ~30%) in peanut EST-SSRs. Such abundance affirmed with earlier reports in other legume crops like chickpea [68], and faba bean [75]. It could be noted that tri-nucleotide motifs are conserved in the genic regions among the legume plants and is also supported by the fact that certain motifs coding for structural proteins are conserved in legumes [76].

In the EST-SSR loci, each tri-nucleotide repeat motif codes a specific amino acid, which plays an important role in various cellular, biological, and metabolic processes in plants [77]. The percentage of tri-nucleotide motifs AAG/CTT, which codes for leucine and lysine was the highest (39.14%) followed by isoleucine and methionine coding repeats ATC/ATG (14.42%). Generally, CG/GC and CCG/CGG are very rare in dicotyledonous plants but common in monocots. In this investigation, out of the 3373 SSRs studied, no GC/CG repeats and only 29 CCG/CGG repeats were found, which is in agreement with the previous results [21,38,58].

Overall, among the total SSR motifs, AG/CT (20.7%) and AAG/CTT (13.25%) were the most abundant motifs followed by ATC/ATG (4.83%), AAT/ATT (4.80%) and AT/TA (4.24%) (S1 Table). However, Koilkonda et al. [21] indicated that AAG/CTT motifs were the most abundant followed by AG/CT motif. In general, AG/CT and AAG/CTT motifs were the most predominant in the earlier reports in peanut [20,78]. Similar patterns of repeat motifs were also observed in other crop like chickpea [68], castor bean [79] and Medicago [69].

The SSR motifs were also classified based on their motifs length [76,80]. Among the total SSRs, 657 (19.47%) SSR motifs were of more than 20 bp (class І) while 2716 (80.53%) were of less than 20 bp (class II) (Table 5). The class ІІ SSRs were present in more numbers than class І, which is in agreement with earlier studies in peanut [14,78]. The frequency of SSR motifs decreased with increase in the length of motifs, indicating negative correlation between frequency and length of motifs. Interestingly, in both class І and class ІІ, tri-nucleotides repeat motifs were detected in higher proportion. In class I microsatellites, the proportion of trinucleotide (266 No) was higher compared to di- (223 No), hexa- (122 No), tetra- (29 No) and penta- nucleotide (17 No). Similarly, in the class II microsatellite, the proportion of tri-nucleotides (876 No) was more than rest of the repeat motifs (Table 5).

**Table 5:** Classification of EST-SSR according to the motif length.

Types of repeats	Class I (>20 bases)	Class II (<20 bases)	Total No. of SSR loci	Average frequency(Kb/SSR)
Dinucleotide	223	705	928	15.20
Trinucleotide	266	876	1142	12.33
Tetranucleotide	29	223	252	55.87
Pentanucleotide	17	450	467	30.15
Hexanucleotide	122	462	584	24.11
**Total**	**657 (19.47%)**	**2716 (80.53%)**	**3373**	**4.17**

### Designing novel primer sets

A total of 2456 primer pairs were designed for 3373 SSR motifs, of which maximum are of tri- (33.92%), followed by di- (20.20%), hexa- (18.69%), penta- (12.66%), compound- (8.35%), and tetra- nucleotides (6.19%). The primers were named by adding the prefix DGR (acronym for Directorate of Groundnut Research) followed by the numbers (S2 and S3 Tables). The remaining SSR containing sequences, either fail to generate primer-pair due to unavailability of flanking site for primer designing or it did not matched the primer designing parameters. Since, EST-SSR markers are generally transferable among distantly related species; therefore, these markers could be used in other *Arachis* species for which little or no information is available on SSRs or ESTs. It is reported that approximately, 96% of the primers designed for *Medicago truncatula* produced amplicons in six other Medicago species, and 66% primers were polymorphic between medicago and ryegrass [81]. Moreover, these markers are also very good candidates for the development of conserved orthologous markers especially for genetic analysis and breeding of minor or poorly-funded crop species including wild *Arachis* species.

#### Sorting of EST-SSRs based on functional relevance and its PCR validation

The genic microsatellites are such class of marker which can target functional polymorphisms within genes and contribute to the ‘direct allele selection’, for any target trait [67]. It could be noted that the SSR motifs are conserved in the genic regions among the legume plants and certain motifs coding for structural proteins are conserved in legumes [76]. Although, SSRs are non-randomly distributed across protein-coding regions, UTRs, and introns [82], but UTRs are predominant in microsatellites than CDS [19,83] and presence of SSRs in the 5’ UTRs are required for gene regulation. Moreover, SSRs in the 3’ UTRs are needed for transcription slippage and expanded mRNA production, which can disrupt splicing and other cellular functions [19]. Role of SSRs in functional genes are not yet properly understood, and further studies are needed to find the effect of microsatellites in gene expression of functional gene containing SSR. However, structural variation in SSR motifs, regulating the gene expression at transcript level and altering the phenotype was reported for amylose content and waxy gene expression [84]. Considering this, it is emphasized that the selection of SSR motifs on the basis of functional relevance to different stresses can be advantageous, so as to get more number of polymorphism in the relevant genotypes. Therefore, the newly designed primers were sorted based on its relevance to various stresses and other functional unigenes containing SSRs (S2 Table).

Among 366 primer pairs, 339 (92.62%) were amplified, which illustrated the precision or suitability of *in silico* primer designing criteria employed for primer designing (Table 6). Within the PCR validated EST-SSRs, 295 (80.6%) and 71 (19.4%) were derived from stress relevant and other functional unigenes respectively. Out of all the synthesized primer-pairs, 39 (10.66%) displayed polymorphism within 11 selected peanut genotypes, of which, 34 and 5 were derived from stress relevant and other functional unigenes respectively (Table 7 and S2 Table). Total number of detected alleles ranged from 1–12 with an average of 3.77 alleles per marker (S2 Table).

**Table 6:** PCR validation of SSRs having functional relevance to various stresses across selected peanut genotypes.

Types of SSRs	No. of primers	No. of primers Amplified	No. of polymorphicprimers	Rang of PIC	Class I	Class II
Dinucleotide	75	66 (88.00%)	10 (13.34%)	0.345–0.375	7	3
Trinucleotide	128	119 (92.96%)	15 (11.72%)	0.028–0.375	4	11
Tetranucleotide	20	20(100%)	02 (10.00%)	0.191–0.365	1	1
Pentanucleotide	53	50 (94.34%)	03 (5.66%)	0.314–0.346	0	3
Hexanucleotide	67	63 (94.03%)	03 (4.48%)	0.251–0.375	1	2
Compound	23	21 (91.30%)	06 (26.09%)	0.139–0.375	NA	NA
**Total**	**366**	**339 (92.62%)**	**39 (10.66%)**	**0.028–0.375**	**13**	**20**

**Table 7:** List of polymorphic primer with predicted function based on sequence homology.

S. No.	Primer name	Motif	Predicted function based on sequence homology	No. of alleles	Range of amplification (bp)	PIC Value
**Stress relevance unigenes containing SSR**						
1	DGR-37	TCT	peroxisome biogenesis protein 19-1-like	8	154–244	0.275
2	DGR-41	TTC	mitogen-activated protein kinase kinase kinase 3-like	6	154–260	0.346
3	DGR-48	CTT	mitogen-activated protein kinase kinase kinase 3-like	2	122–186	0.375
4	DGR-52	GGC	alternative oxidase	4	133–158	0.305
5	DGR-58	AAG	hydroxyproline-rich glycoprotein family	6	118–140	0.375
6	DGR-87	GAATT	aquaporin pip2-7	6	120–172	0.346
7	DGR-105	CA	malate dehydrogenase	4	152–198	0.375
8	DGR-114	TTC	wound-responsive family protein	12	154–372	0.351
9	DGR-128	AGTG	ethylene response protein	6	139–526	0.191
10	DGR-162	(ATT)6 (ATT)5	abscisic acid 8-hydroxylase	4	134–168	0.139
11	DGR-163	AGA	abscisic acid 8-hydroxylase	4	164–240	0.139
12	DGR-166	TTC	alcohol dehydrogenase-like protein	4	148–173	0.311
13	DGR-171	TAT	oxidation resistance protein	6	158–196	0.351
14	DGR-172	TTC	proline-rich family protein	6	148–170	0.375
15	DGR-174	(CAA)5 (GCA)7	hydroxyproline-rich glycoprotein family protein	6	160–285	0.370
16	DGR-179	(AG)7 (GA)8 (AG)6	heat stress transcription factor b-3-like	3	112–142	0.346
17	DGR-198	TTTTTC	casein kinase family protein	4	128–161	0.375
18	DGR-203	TC	gaba receptor-associated	3	257–284	0.346
19	DGR-216	AAG	3-epi-6-deoxocathasterone 23-monooxygenase-like	6	162–229	0.339
20	DGR-253	TA	glutamine synthetase	3	114–142	0.346
21	DGR-258	CT	lrr receptor-like serine threonine-protein kinase fei 1-like	6	137–153	0.375
22	DGR-282	AG	syntaxin-61-like	3	144–165	0.346
23	DGR-289	AG	tubby-like f-box protein 8-like	5	87–110	0.365
24	DGR-304	CT	ankyrin repeat domain-containing protein 13c-b-like	5	182–302	0.365
25	DGR-308	TC(8)	ankyrin repeat-rich protein	4	121–174	0.305
26	DGR-312	CT	c2h2-like zinc finger protein	4	152–172	0.375
27	DGR-316	TTTG	dof zinc finger	5	190–287	0.365
28	DGR-322	TCCAAC	ethylene-responsive transcription factor crf4-like	3	160–179	0.251
29	DGR-329	AGATC	gdsl esterase lipase at1g29670-like	6	243–374	0.314
30	DGR-335	AGA	heat shock protein sti-like	6	145–178	0.375
31	DGR-338	ATT	hypoxia-responsive family protein	4	100–153	0.028
32	DGR-361	(CT)7 (TCT)5	probable xyloglucan endotransglucosylase hydrolase protein 28-like	3	135–150	0.346
33	DGR-362	CTT	probable xyloglucan endotransglucosylase hydrolase protein 30-like	6	261–382	0.375
34	DGR-386	CTCAAT	vicilin 47k	7	240–371	0.370
**Other functional unigenes containing SSR**						
35	DGR-146	AAGAG	senescence-inducible chloroplast stay-green protein	6	185–255	0.346
36	DGR-259	(ATA)6 (GA)7	mads transcription factor	4	110–148	0.375
37	DGR-263	TC	pfkb-type carbohydrate kinase family protein	6	160–243	0.372
38	DGR-294	TCT	udp-galactose transporter 1-like	8	138–186	0.305
39	DGR-301	(CCT)5 (TCT)7	alpha beta-hydrolases superfamily protein	5	154–271	0.356

On the similar lines, Peng et al. [85] have also observed 89.2% primer amplification and 6.5% polymorphism in their EST derived SSR marker set. Comparatively, in the present study, on an average higher number of alleles were recorded. Pandey et al. [14] also got nearly similar results for allele numbers, which ranged from 2 to 14 with an average of 3.2 alleles in cultivated peanut. These results also corresponded well with other studies in peanut, where 2.3 [31], 2.44 [30] and 2.99 [86] alleles per marker is reported. A total of 384 AG/CT di-nucleotide motifs were utilized for primer designing, of which 327 (85%) were having functional annotations. Among these, 55 primers with functional enrichment were synthesized and validated on 11 diverse genotypes, resulted in 8 polymorphic primers (S2 Table).

Among the 39 polymorphic markers, 2–12 alleles amplified with average of 5.1 alleles per marker. The PIC values of polymorphic primers ranged from 0.028 to 0.375 with an average of 0.325 (Table 7). In general, the PIC values of less than 0.5 is also reported for the EST-SSR markers developed by other research groups in cultivated peanut [14,87], which is evident of low level of polymorphism in those genotypes. It has also been well documented that EST-SSRs are less polymorphic than genomic SSRs because of greater DNA sequence conservation in transcribed regions [14,88]. On the basis of their sequence homology with stress relevant genes, the polymorphic EST-SSR primers showing high PIC values (in parenthesis) like DGR-48 (0.375), DGR-58 (0.375), DGR-105 (0.375), DGR-172 (0.375), DGR-174 (0.370), DGR-198 (0.375), DGR-258 (0.375), DGR-289 (0.365), DGR-316 (0.365), DGR-362 (0.375) and DGR-312 (0.375) could be further validated on mapping population. However, two EST-SSR markers *viz*., DGR-259 (0.375) and DGR-263 (0.372) associated with the other functional genes have also shown higher degree of polymorphism (Table 7).

## Conclusions

In peanut, a constant increase in the volume of sequence data generated from EST projects running in different labs across the world has facilitated the identification of a large number of genic SSRs. During recent years, a wealth of genomic data has been generated in peanut by high throughput transcriptome sequencing [25,63,85,89,90]. Besides, EST database are equally informative and consequential as high throughput transcriptome data. The present work offers complete utilization of EST database for development of SSR markers exclusively from cultivated peanut.

The information of polymorphic EST-SSRs markers not only facilitated better understanding the nature of SSRs in the peanut genome, but also provided a useful source for conducting additional genetic and genomic studies to improve this crop [58]. As demonstrated by the functional annotation, these polymorphic EST-SSR markers increased the chances of linkage to loci, contributing to stress tolerance or resistance. Because of their association to the coding regions, these polymorphic markers could be further validated on mapping population segregating for various biotic and abiotic stress-tolerance or resistance traits. As these genic EST-SSRs are more likely to be conserved between closely related species, they can also facilitate better cross genome comparisons [91]. The most noticeable feature of EST-SSRs is its transferability in related species which makes it potentially more useful for comparative mapping studies [67]. These markers could be also employed in characterizing related legume genomes, with no prior available information. There is a need to validate all the developed EST-SSRs markers, for polymorphism, so as to enhance the density of the existing genetic maps of peanut. In the longer run, development of allele-specific markers for the genes controlling various biotic and abiotic traits will be important for QTL mapping and marker-assisted selection in peanut improvement.

To sum up, this study reports the primer sequences for 2456 novel EST-SSR markers, and analysis of 366 markers, selected on the basis of stress related functions, on a set of 11 diverse genotypes, identified 39 polymorphic markers. It is hoped that the identified EST-SSR markers will not only enrich the current marker resources but also benefit the international peanut research community working on molecular breeding.

## Supporting Information

S1 Fig Representative gel photograph showing amplification of 4 DGR primers (a. DGR- 41, b. DGR- 114, c. DGR-146 and d. DGR-179) in 11 peanut genotypes. Where M: 50 bp DNA marker, 1: GPBD-4, 2: JSP-39, 3: R-2001-3, 4: ALR-2, 5: VG-09405, 6: ICGV 86590, 7: CS-85, 8: CS-319, 9: JL-24, 10: GG-20, 11: TG 37-A
(TIF)

S1 Table Overall frequency distribution of 3373 motif repeats identified from *A*. *hypogaea* unigenes. (XLSX)

S2 Table PCR validation of 366 EST-SSR primers, using the panel of 11 peanut genotypes. (Where light-green and pink color codes represent stress-relevant and other functional unigenes respectively)
(XLSX)

S3 Table A set of 2456 novel EST-SSR primers, designed from publically available *Arachis hypogaea* databases. (Excluding 366, stress relevant and other functional unigenes containing SSR markers used for the PCR validation)
(XLSX)

S1 Script Search for sequence similarity of primer sequence (query) from EST database (Subject). (PL)

S2 Script Extraction of specific sequences based on sequences ID. (PL)
