# Correction: Novel and Stress Relevant EST Derived SSR Markers Developed and Validated in Peanut

**DOI:** 10.1371/journal.pone.0133537

**Published:** 2015-07-15

**Authors:** The PLOS ONE Staff

There are errors in the Author Contributions. The correct contributions are: Conceived and designed the experiments: RT TCB. Performed the experiments: GPM TCB. Contributed reagents/materials/analysis tools: JRD TCB. Wrote the manuscript: TCB GPM.

The publisher apologizes for the error.
