# Supplementary figures and images for: Novel and Stress Relevant EST Derived SSR Markers Developed and Validated in Peanut
Source: PLoS One. 2015 Jun 5;10(6):e0129127. doi: 10.1371/journal.pone.0129127 (PMC4457858; doi:10.1371/journal.pone.0129127)

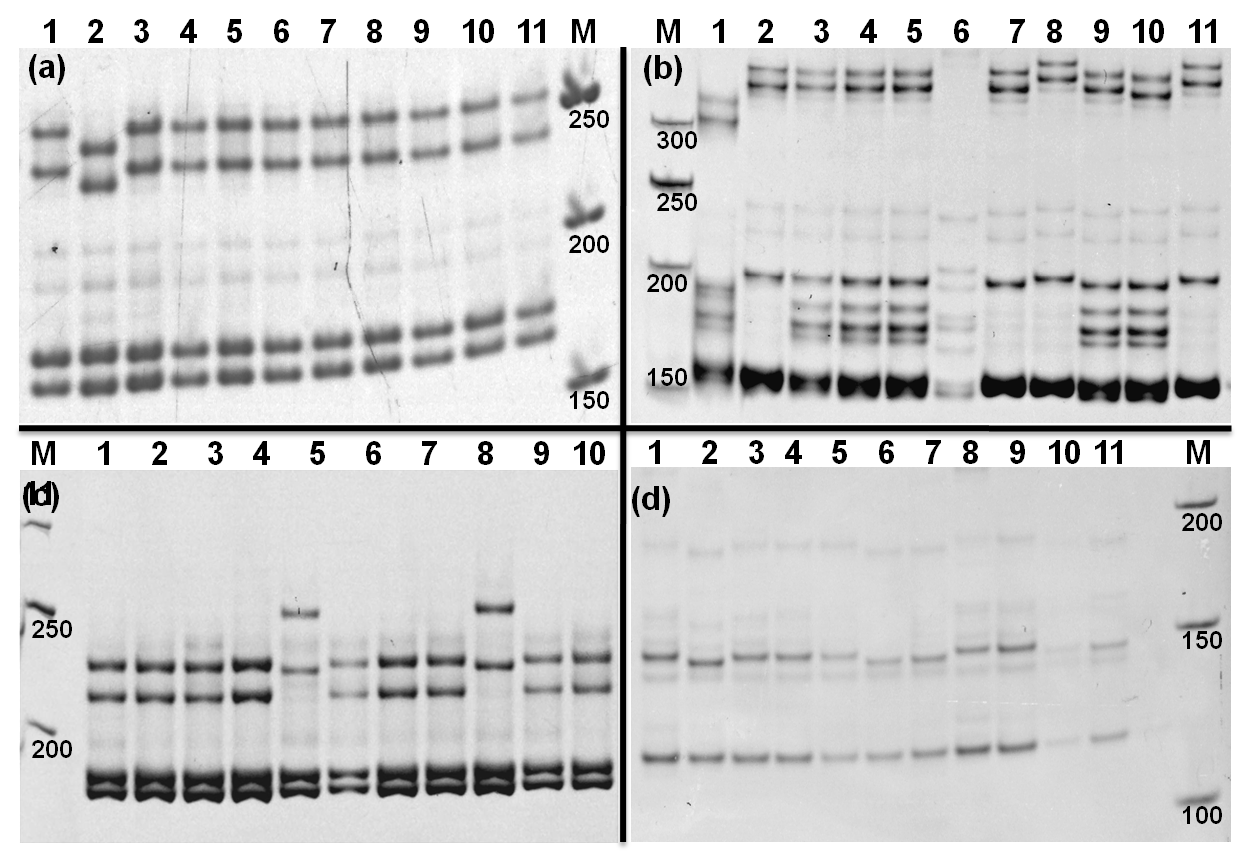

Supplement: S1 Fig — Where M: 50 bp DNA marker, 1: GPBD-4, 2: JSP-39, 3: R-2001-3, 4: ALR-2, 5: VG-09405, 6: ICGV 86590, 7: CS-85, 8: CS-319, 9: JL-24, 10: GG-20, 11: TG 37-A (TIF) [file pone.0129127.s001.tif]
